# Frequency of actionable secondary findings in 7472 Korean genomes derived from the National Project of Bio Big Data pilot study

**DOI:** 10.1007/s00439-023-02592-8

**Published:** 2023-09-20

**Authors:** Youngjun Kim, Jeong-Min Kim, Hye-Won Cho, Hyun-Young Park, Mi-Hyun Park

**Affiliations:** 1 Division of Genome Science, Department of Precision Medicine, National Institute of Health, Cheongju, Republic of Korea; 2 Department of Precision Medicine, National Institute of Health, Cheongju, Republic of Korea

## Abstract

Exome and genome sequencing (ES/GS) in genetic medicine and research leads to discovering genomic secondary findings (SFs) unrelated to the purpose of the primary test. There is a lack of agreement to return the SF results for individuals undergoing the test. The aim of this study is to investigate the frequency of actionable secondary findings using GS data obtained from the rare disease study and the Korean Genome and Epidemiology Study (KoGES) in the National Project of Bio Big Data pilot study. Pathogenic (P) or likely pathogenic (LP) variants of 78 SF genes recommended by the American College of Medical Genetics and Genomics (ACMG) were screened in the rare disease study and KoGES. The pathogenicity of SF gene variants was determined according to the ACMG interpretation. The overall SF rate was 3.75% for 280 individuals with 298 P/LP variants of 41 ACMG SF genes which were identified among 7472 study participants. The frequencies of genes associated with cardiovascular, cancer, and miscellaneous phenotypes were 2.17%, 1.22%, and 0.58%, respectively. The most frequent SF gene was *TTN* followed by *BRCA2*. The frequency of actionable SFs among participants with rare disease and general population participants in the Korean population presented here will assist in reporting results of medically actionable SFs in genomic medicine.

**Supplementary Information:**

The online version contains supplementary material available at 10.1007/s00439-023-02592-8.

## Introduction

Exome and genome sequencing (ES/GS) are rapidly integrated into medicine as well as healthcare research globally owing to the decreasing cost of sequencing and advances in bioinformatics tools (Stark et al. 2019; Van El et al. 2013). Consequently, ES/GS have become crucial process in mainstream medicine and healthcare systems, which contributes to precision medicine and improving the health of various populations (Suwinski et al. 2019). The use of ES/GS analysis in a clinical context such as genetic diagnosis of rare diseases and cancers may potentially identify genomic incidental/secondary findings (SFs) from the patients or their family regardless of the primary test’s purpose (Green et al. 2013).

Genomic incidental findings/SFs represent major issues in clinical sequencing in terms of the range of the findings and the manner of reporting results to the patient/family or study participants. The American College of Medical Genetics and Genomics (ACMG) has published a recommendation for reporting incidental/SFs (Green et al. 2013), which provides a minimal list of clinically actionable genes to actively screen for pathogenic (P) or likely pathogenic (LP) variants in clinical ES/GS. The actionability of genes was reviewed by the Secondary Finding Working Group in ACMG and 78 actionable genes have been reported (version 3.1) (Miller et al. 2022). Based on the ACMG SF recommendation, identifying a pathogenic variant in the SF genes may represent an opportunity for enabling early intervention to prevent the development of SF-related diseases in individuals that have undergone clinical sequencing, although there may be additional ethical considerations reporting results depending on the individual medical circumstances (Venner et al. 2022; Zawatsky et al. 2021).

Several studies have attempted to identify SFs across diverse studies and populations such as the NIH Undiagnosed Diseases Program (Lawrence et al. 2014), 1000 Genomes Project (Olfson et al. 2015), Qatar genome program (Elfatih et al. 2021a), eMERGE network participants (Gordon et al. 2020), and DISCO study (Huang et al. 2022) representing the frequency of SFs from 0.59 to 17% (Elfatih et al. 2021b). However, previous studies using ES/GS analysis were mainly performed in the western population; few studies have analyzed other populations, particularly in Asia (Chetruengchai and Shotelersuk 2022; Horiuchi et al. 2021; Landry et al. 2018; Pan and Xu 2020; Sirugo et al. 2019). Two studies have reported the screening of SFs in the Korean population by analyzing 196 and 1303 individuals’ whole-exome data from study participants, in which the SF rates have been found to be 6.63% and 2.46% respectively (Jang et al. 2015; Kwak et al. 2017). Previous SF analyses have reported contrasting SF rates despite evaluating the same ACMG SF 56 genes, which may be affected by differences in sample size, variant filtering criteria, and database used between studies (Elfatih et al. 2021b).

The National Project of Bio Big Data is a national project that aims to implement precision medicine and national health promotion; it has generated GS data, clinical information, and lifestyle data of the Korean population (KISTI 2021). In the rare disease study of this project, patients with rare diseases and their families were recruited for genetic diagnosis as well as for finding new genetic factors related to rare disorders based on the GS analysis. Additionally, the Korean Genome and Epidemiology Study (KoGES), another study in the project, generated GS data for a general population.

The aim of the present study is to identify clinically actionable variants of 78 ACMG SF genes and to investigate the frequency of SFs in 7472 Korean genomes obtained from the two studies, the rare disease study and the KoGES of the National Project of Bio Big Data pilot study.

## Materials and methods

### Study population

The pilot study of the National Project of Bio Big Data (KISTI 2021) consisted of independent studies including the rare disease study and KoGES. Genetic analyses were performed for 4972 genomes and 2500 genomes obtained from the rare disease study and KoGES, respectively. The rare disease study participants consisted of singleton proband, duo, trio, and more than trio families. The rare diseases were classified into 19 disease categories including cardiovascular disorder, neurodevelopment disorder, congenital disorder, metabolic disorder, tumor syndrome, and so on (Supplementary Table 2). Basic information including age, sex, and ethnicity, as well as clinical information including disease, medical history, family history, and phenotypes with HPO term were input by a clinician. The KoGES was a general population-based study that recruited community dwellers aged > 40 years at the baseline examination from two locations, Ansan and Ansung (Kim et al. 2017). A total of 7472 participants including 2186 probands, 2786 families (without probands) in the rare disease study, and 2500 KoGES participants were analyzed in the present study after they provided informed consent (Supplementary Table 1).

### Genome sequencing data

GS data was generated using samples obtained from all study participants in the National Project of Bio Big Data pilot study (https://www.cirn.re.kr). GS data were obtained using identical methods with standard operating procedures across the studies. Briefly, genomic DNA was extracted from peripheral whole blood of the study participants, followed by sequencing conducted on the Illumina NovaSeq 6000 platform with an average of 30-read coverage. FASTQ files were aligned to the human reference genome GRCh38 using BWA software (v0.7.15-r1140). Variant calling was performed using Genome Analysis Toolkit (GATK, Broad Institute) based on Apache Spark (v4.2.4.1). Joint genotype calling was performed on individual gVCF files to improve the validity of the variants using a GATK joint genotyping pipeline to generate a joint-multi-sample VCF file. Variant quality score recalibration (VQSR) was performed to filter variants. All procedures were performed as described previously (https://www.kobic.re.kr/ngp/pipeline).

### Variant classification

**Fig. 1:**
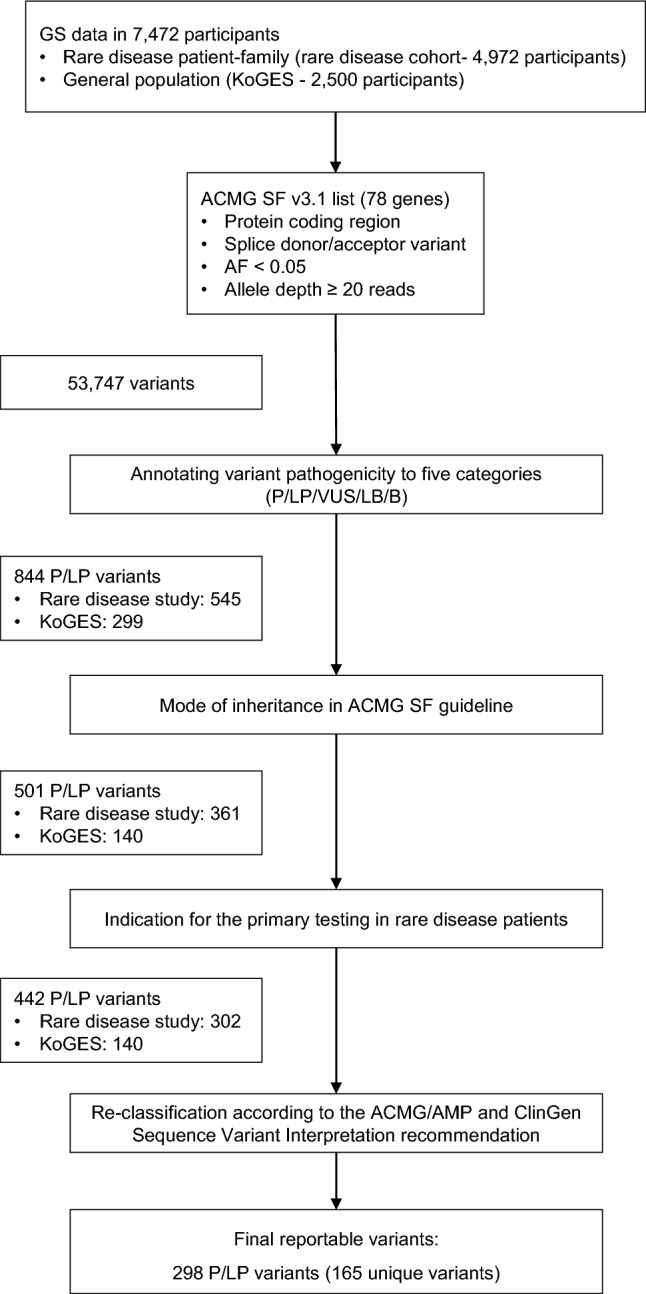
Flow chart for screening pathogenic/likely pathogenic variants in actionable secondary finding genes from the ACMG SF list v3.1. *GS* genome sequencing, *KoGES* Korean Genome and Epidemiology Study, *AF* allele frequency, *P/LP* pathogenic/likely pathogenic

The variants obtained using the PASS filter after the filtering step were annotated using the Ensembl Variant Effect Predictor (McLaren et al. 2016) and ANNOVAR (Wang et al. 2010). The variants of 78 ACMG SF genes were included only for the protein coding region or splice variants, and these had an allele frequency of < 0.05 (gnomAD) and a minimum coverage of 20 reads (Fig. 1). The variants were classified into five categories, pathogenic (P), likely pathogenic (LP), likely benign (LB), benign (B), and uncertain significance (VUS), according to the ACMG variant interpretation guidelines (Richards et al. 2015) using bioinformatics software (Li and Wang 2017; Seo et al. 2020; Xiang et al. 2020), followed by considering the mode of inheritance in the ACMG SF genes associated with the phenotype and primary test indication of probands in rare disease study participants. We then reclassified the P and LP variants manually considering the ACMG criteria and ClinGen Sequence Variant Interpretation recommendations (Abou Tayoun et al. 2018; Biesecker and Harrison 2018; Brnich et al. 2020; Ghosh et al. 2018; Pejaver et al. 2022; Rehm et al. 2015; Tavtigian et al. 2018; Tavtigian et al. 2020).

Primary finding was defined as the P/LP variants of the ACMG SF gene relevant to the diagnostic indication of the patient for the purpose of sequencing (Green et al. 2013). Based on the definition, the patients with rare diseases who carried the P/LP variant of the SF genes were not considered to show SFs when their SF genes were relevant to the primary test phenotype. In addition, the individuals with monoallelic P/LP variants of SF genes related to autosomal recessive inheritance in the ACMG SF gene associated with phenotypes were identified, but not reported as SF. The SNV/indels variants were assessed in the analysis, but structural variants were not included in our analysis.

## Results

### Demographics of study population

A total of 7472 study participants included 4972 individuals from the rare disease study and 2500 individuals from the KoGES. Participants from the rare disease study comprised 669 singleton (13.5%), 590 duo (11.9%), 3309 trio (66.6%), 374 quartet and quintet (7.5%), and 30 others (1.1%) (Supplementary Table 1). More than half of the participants from the rare disease study comprised trio, quartet, or quintet families (*n* = 3683, 74.1%). The mean age of the participants from the rare disease study were 21.5 ± 20.4 years for patients and 42.6 ± 10.4 years for family members. Participants from KoGES had a mean age of 51.8 ± 8.3 years at baseline examination (Supplementary Table 1). Among the genomes of the 7472 participants, 298 pathogenic/likely pathogenic variants of the 78 ACMG genes were filtered (Fig. 1).

### Overall rate of SFs

**Fig. 2:**
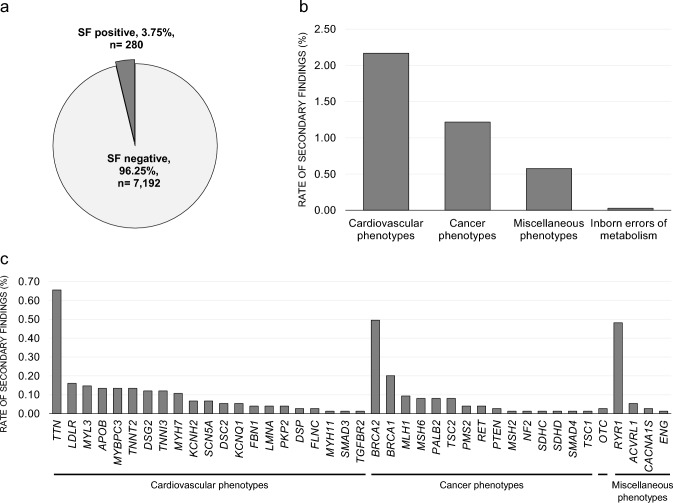
Secondary finding rate in the study participants. **a** SF rate among the total participants. **b** SF variant rate according to phenotypes. **c** SF variant rate according to genes. *SF* secondary finding; the *OTC* is related to the inborn errors of metabolism phenotypes

Among the 7472 study participants, 280 individuals carried 298 P/LP variants (165 unique variants) of ACMG SF genes (3.75%) (Fig. 2a). The genes associated with cardiovascular phenotypes were the most frequent (2.17%) followed by those associated with cancer (1.22%), miscellaneous (0.58%), and inborn errors of metabolism (0.03%) in the two studies (Fig. 2b). The frequency of the findings based on the subcategory of the phenotypes, pathogenic variant, and studies are shown in the Supplementary Table 3. A total of 41 genes among the 78 ACMG genes included the P/LP variants (Supplementary Table 3); the most frequent SF gene was *TTN* (0.66%), followed by *BRCA2* (0.50%) and *RYR1* (0.48%) (Fig. 2c). Ten variants were identified commonly in more than five participants; the most frequent variant was c.10819G>T in *TTN* (n = 14) followed by c.170C>G in *MYL3* (n = 11) and c.452G>A in *TNNT2* (n = 10) (Table 1 and Supplementary data). Also, there were 17 participants who show double heterozygosity and 1 participant who have the triple pathogenic variants.

**Table 1:** Pathogenic/likely pathogenic variants identified in five or more participants among the study participants

Gene	Coding DNA change	Protein change	Variant class	Type	No. of participants	Rare-patient	Rare-family	KoGES
*TTN*	c.10819G>T	p.Glu3607Ter	Stop gained	LP	14	5	3	6
*MYL3*	c.170C>G	p.Ala57Gly	Missense	LP	11	3	4	4
*TNNT2*	c.452G>A	p.Arg151Gln	Missense	LP	10	5	5	–
*RYR1*	c.1187A>C	p.Glu396Ala	Missense	LP	7	3	3	1
*RYR1*	c.1186G>T	p.Glu396Ter	Stop gained	P	7	3	3	1
*TNNI3*	c.434G>A	p.Arg145Gln	Missense	P	7	2	2	3
*BRCA2*	c.1399A>T	p.Lys467Ter	Stop gained	P	6	2	3	1
*BRCA2*	c.5576_5579del	p.Ile1859LysfsTer3	Frameshift	LP	5	2	3	–
*BRCA1*	c.3627dup	p.Glu1210ArgfsTer9	Frameshift	LP	5	1	1	3
*KCNH2*	c.1474C>T	p.His492Tyr	Missense	LP	5	1	2	2

*KoGES* Korean Genome and Epidemiology Study, *Rare-family* family from rare disease study, *Rare-patient* participant from rare disease study

### SF rate across the two studies

The overall SF rates differ significantly based on the three groups in the two studies, rare disease study and KoGES (Table 2 and Supplementary Fig. 1a). Regarding the SF genes of phenotypes, the SF rate of genes associated with cardiovascular phenotypes was slightly lower in the rare disease family group (without probands, 53/2786, 1.90%) and in KoGES (52/2500, 2.08%) than in the rare disease patient group (57/2186, 2.61%). Further, the SF rate of genes associated with cancer phenotypes was lower in the KoGES (22/2500, 0.88%) than in the rare disease family group (39/2786, 1.40%) and in the rare disease patient group (30/2186, 1.37%). The SF rate indicated that the proportion of pathogenic variants of *TTN* and *LDLR* was similar or higher in the KoGES compared with that in the rare disease study (Supplementary Fig. 1b).

**Table 2:** Secondary finding frequency according to the study participants

	Rare-patient	Rare-family	KoGES
No. of participants	2186	2786	2500
Secondary findings (%)	96 (4.39)	106 (3.80)	78 (3.12)
Related phenotypes (%)			
Cardiovascular	57 (2.61)	53 (1.90)	52 (2.08)
Cancer	30 (1.37)	39 (1.40)	22 (0.88)
Inborn errors of metabolism	1 (0.05)	1 (0.04)	–
Miscellaneous	16 (0.73)	20 (0.72)	7 (0.28)

*Rare-family* family from rare disease study, *Rare-patient* participant from rare disease study, *KoGES* Korean Genome and Epidemiology Study

### Pathogenic variants shared between patients and parents in the rare disease study

**Fig. 3:**
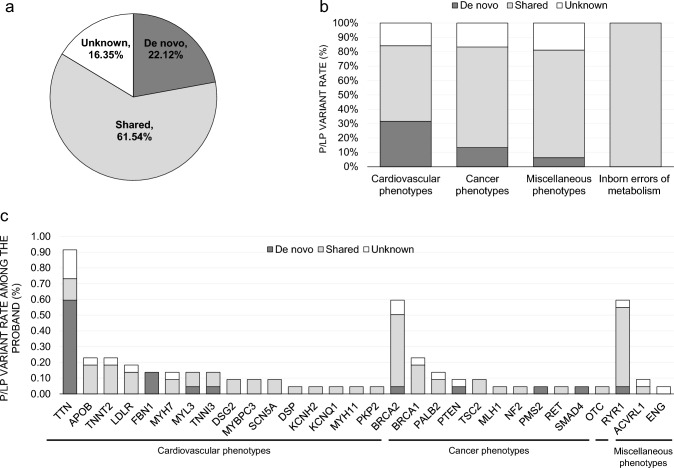
Frequency of shared and de novo secondary finding pathogenic variants among rare disease study probands in trio/quartet/quintet families. **a** Overall P/LP variant frequency. **b** P/LP variant frequency of related phenotypes. **c** P/LP variant frequency of the SF genes. *SF* secondary finding; Unknown, P/LP variants of the probands in singleton or duo family. The *OTC* is related to the inborn errors of metabolism phenotypes

Among the participants of the rare disease patient group, we investigated the frequency of SF variants shared between participants and their parents and de novo variants (Fig. 3a). Among the patients with trio/quartet/quintet families, 61.54% P/LP variants (n = 64/104) were shared with their parents, and there were 22.12% de novo P/LP variants (n = 23/104) and 16.35% were unknown (17/104). In the cardiovascular phenotype, 52.63% of P/LP variants were shared (n = 30/57) and 31.58% were de novo variants (n = 18/57). In the cancer phenotypes, 70.00% of SF variants were shared (n = 21/30) and 13.33% were de novo variants (n = 4/30). In the miscellaneous phenotypes, 75.00% of SF variants were shared (n = 12/16) and 6.25% were de novo variants (n = 1/16) (Fig. 3b). According to the SF genes, the de novo variants were identified in *TTN*, *FBN1, MYL3, TNNI3, BRCA2, PTEN, PMS2, SMAD4,* and *RYR1* in our study population (Fig. 3c, Supplementary data).

### Carrier status findings in autosomal recessive disorders

We identified participants who carried P/LP variants of ACMG genes related to autosomal recessive disorders; however, all P/LP variants in participants had a heterozygous status in the present study. The P/LP variants of 6 genes out of 8 genes related to autosomal recessive disorders in ACMG genes were identified; the most frequent gene was *ATP7B* (1.24%) followed by *GAA* (0.32%) and *MUTYH* (0.31%) (Supplementary Fig. 2).

## Discussion

In this present study, the overall SF rate was 3.75% including individuals carrying the P/LP variant of clinically actionable genes included in the latest version (3.1) of the ACMG SF list. A previous SF analysis of a Korean population reported an SF rate of 6.6% (Jang et al. 2015), which was considerably higher than that reported in the present study. This difference could arise from several factors, including the fact that the previous study population included candidate patients with Mendelian diseases; however, the study did not exclude individuals with a primary indication for the test because there were no phenotype data in the analysis (Jang et al. 2015). Further, the study used a different clinical database (HGMD) to classify P/LP variants as SFs compared to that used in the present study. Also, the SF rate showed 2.64% of study participants even though the variants based on the ClinVar database (P/LP in ClinVar) (Supplementary Fig. 3). Moreover, the SF rate in the present study was in agreement with findings of a recent review that reported varying frequencies of incidental findings in the range of 0.5–17% (Elfatih et al. 2021b). We excluded patients carrying the P/LP variant of SF gene associated with phenotypes corresponding to the primary test indication. The primary findings of patients might have been missed owing to the lack of phenotype information of the study participants; it was likely that the SF rate was overestimated in the analyses (Biesecker 2016). To moderate the overestimation in the rare disease study group, patients were excluded when their phenotypes were included in one of four broad categories: cancer, cardiovascular disorder, inborn errors of metabolism, and miscellaneous disorders. Although the actionability of the ACMG SF genes (v3.1) may differ between the Korean population and other ethnic populations based on the characteristics of study participants such as different penetrance and genetic background. Our results can help preparing clinical guidelines for reporting results of SFs to the Korean population.

The ACMG SF list has not been validated for screening the general population. However, the ACMG SF genes can support continued research and discussions regarding the factors to consider in population screening programs. Such factors include penetrance and genotype–phenotype correlations to examine the efficacy of using such genomic screening in asymptomatic individuals, and the SFs may provide an opportunity to identify a potentially life-threatening genetic risk factor (Miller et al. 2021b). A recent study showed that the disability-adjusted life years (DALYs) of an individual harboring rare variants of ACMG genes are higher than those of individuals that have relatively more common variants (Jukarainen et al. 2022). Thus, the results can help explain the DALYs attributable to the presence of a deleterious rare variant of ACMG genes which has a considerable impact on healthy life years. In the present study, we investigated the lipid profile at baseline examination among the KoGES participants who carried the pathogenic variant of *LDLR* (Supplementary Table 4). The participants showed a high lipid profile; however, the lipid level in most participants at the final examination exceeded the criterion for dyslipidemia; thus, further analysis is required in follow-up studies.

*TTN* had the most frequent pathogenic variant among the 78 SF genes in our results. *TTN* was included in the SF list at v3.0 for *TTN* truncating variants (TTNtvs) alone. The frequency of TTNtvs has been reported to be 0.5–1% in a previous large population study (Miller et al. 2021a); we found a similar frequency of TTNtvs (0.66%). In a previous study, TTNtvs have been associated with clinical phenotypes such as increasing left ventricular size (Haggerty et al. 2019). Moreover, individuals of African ancestry show a relatively weaker association between TTNtvs and dilated cardiomyopathy; thus, follow-up of the cardiovascular symptom/phenotype in individuals with TTNtvs is needed in our population. This will benefit participants and help prepare guidelines to improve the utility of *TTN* in clinical settings. In previous SF studies performed in Asian populations, the frequency of the *TTN* variant has been found to be 1.2% in the Chinese population (Huang et al. 2022); a large-scale Asian genomics study comprising three populations reported *TTN* carrier frequencies of 0.60% in Chinese, 0.67% in Indian, and 0.44% in Malay populations (Chan et al. 2022). The most frequent variant in the present analysis, c.10819G>T in *TTN,* showed an allele frequency of 0.0005778 in the East Asian population; however, the overall allele frequency was 0.00001971 (gnomAD v3.1). The variant was more frequent in East Asian populations than in European populations. In the present study, the frequency of the variant was 0.0019, which is considerably higher than that reported in the public database. Although the variant was an LoF variant, there are no report on the pathogenicity of the variant in ClinVar or other databases. Thus, further analyses such as penetrance of the variant and functional studies are needed to validate the pathogenicity of the variant in our population.

Among the rare disease study group, we investigated whether the SF P/LP variants were shared between participants and their parents or were de novo variants; more than half of the P/LP variants in the participants were shared with at least one of their parents. Identifying these shared P/LP variants can provide opportunities for early diagnosis, medical management, or effective clinical intervention to the participants and their parents or other family members who did not exhibit the related disease phenotypes despite a lack of sufficient clinical information of the participants (Miner et al. 2022; Thompson et al. 2018).

All identified SF genes were related to autosomal dominant disorders according to the ACMG SF list; however, there were several participants carrying the P/LP variants with heterozygous status for the autosomal recessive disorders related to genes such as *ATP7B* and *MUTYH* (Supplementary Fig. 2). The ACMG guidelines recommend reporting pathogenic bi-allelic variants only in autosomal recessive disorder-related genes; we did not include them as actionable SFs in the present study. The burden of analysis would have increased when investigating the P/LP variants of autosomal recessive disorder-related genes with heterozygous status, and carrier screening and discovery efforts would have increased Sanger sequencing validation costs and the time required for genetic counselors and medical geneticists to report results (Green et al. 2013). However, the carrier status of the study participants was investigated in our SF screening; it may provide valuable information concerning their offspring or for planning children. In addition, an analysis was performed regarding the Wilson disease that is associated with *ATP7B* homozygous mutation showing a prevalence of 38.7 per million people in Korea (Choe et al. 2020). Thus, screening the P/LP variant of *ATP7B* and even carrier status may be beneficial to the Korean population; however, it needs extensive evaluation before reporting the results to the study participants.

There were several limitations in the present study. First, the size of our project was small compared to that of other large-scale projects conducted in the western population (All of Us Research Program et al. 2019; Gordon et al. 2020; Van Hout et al. 2020). However, our study size was comparatively large for an Asian population (Huang et al. 2022; Yamaguchi-Kabata et al. 2018), particularly in terms of single national population (Chan et al. 2022; Jang et al. 2015; Kwak et al. 2017), as well as other populations (Elfatih et al. 2021a; Rodríguez-Salgado et al. 2022). Second, we could not determine the exact penetrance of the P/LP variants in our study population because participants who carried the P/LP variant could not be followed. The penetrance likely differs based on ethnicity (Forrest et al. 2022); thus, further analyses are required to evaluate the penetrance of the P/LP variants in our population with large-scale follow-up studies. Also, this study is to investigate the frequency of secondary finding in the study participants, not to return the SF results to the study participants. Thus, the study participants could not obtain the SF results, genetic counseling about SF. Lastly, we assessed SNV and indel only, not the structural variations, large rearrangements or exon-range alteration even using the genome data. There might be the pathogenic structural variants of SF genes, thus, further analyses will be needed.

In summary, we determined a rate of actionable SFs of 3.75% among participants in the two studies: rare disease and KoGES according to the ACMG SF gene list v3.1. The most frequent gene associated with disease domain was cardiovascular phenotypes (2.17%) followed by cancer phenotypes (1.22%) and miscellaneous phenotypes (0.58%). The most frequent SF gene was *TTN* (0.66%). We found that the frequencies of pathogenic variants of actionable SF genes differed to a minor extent between the general population and rare disease group family–patient population. Our findings can help evaluate the clinical SF guidelines for the general population and patient–families with rare diseases who underwent genome sequencing analysis.

### Supplementary Information

Below is the link to the electronic supplementary material.

Supplementary file1 (DOCX 67 KB)

Supplementary file2 (XLSX 25 KB)

## Data Availability

The datasets generated during and/or analysed during the current study are available in the National Project of Bio Big Data pilot study repository (https://www.cirn.re.kr) with permission.
