# Supplementary material for: Frequency of actionable secondary findings in 7472 Korean genomes derived from the National Project of Bio Big Data pilot study
Source: Hum Genet. 2023 Sep 20;142(11):1561–9. doi: 10.1007/s00439-023-02592-8 (PMC10602966; doi:10.1007/s00439-023-02592-8)
Supplement: Supplementary file 1 — Supplementary file1 (DOCX 67 KB) [file 439_2023_2592_MOESM1_ESM.docx]

**Supplementary Table 1** Demographic information about the study groups

|  | Total |  | Rare-patient | Rare-family | KoGES |
| --- | --- | --- | --- | --- | --- |
| No. of participants | 7,472 |  | 2,186 | 2,786 | 2,500 |
| Age (years) |  |  |  |  |  |
| Mean | 39.5±18.3 |  | 21.5±20.4 | 42.6±10.4 | 51.8±8.3 |
| Median | 43 |  | 14 | 41 | 50 |
| Sex (%) |  |  |  |  |  |
| Men | 3,701 (49.5) |  | 1,161 (53.1) | 1,312 (47.1) | 1,228 (49.1) |
| Women | 3,771 (50.5) |  | 1,026 (46.9) | 1,473 (52.9) | 1,272 (50.9) |
| Family structure |  |  |  |  |  |
| Singleton | 669 (13.5) |  | 669 (30.6) | - | - |
| Duo | 590 (11.9) |  | 304 (13.9) | 286 (10.3) | - |
| Trio | 3,309 (66.6) |  | 1,103 (50.4) | 2,206 (79.2) | - |
| More than trio | 374 (7.5) |  | 110 (5.3) | 264 (9.5) | - |
| Others | 30 (0.4) |  |  | 30 (1.1) |  |

Others consisted of one proband and two siblings, or sibling only

**Supplementary Table 2** Disease categories in rare disease study

| Disease category | Cases (%) |
| --- | --- |
| Cardiovascular disorders | 297 (11.6) |
| Ciliopathies | 15 (0.6) |
| Dermatological disorders | 31 (1.2) |
| Dysmorphic or congenital abnormality | 120 (4.7) |
| Endocrine disorders | 66 (2.6) |
| Gastroenterologic disorders | 67 (2.6) |
| Growth disorders | 95 (3.7) |
| Haematologic or immunologic disorders | 113 (4.4) |
| Hearing or ear disorders | 28 (1.1) |
| Infectious diseases | 3 (0.1) |
| Metabolic disorders | 57 (2.2) |
| Neurologic or neurodevelopmental disorders | 1,019 (39.8) |
| Ophthalmologic disorders | 108 (4.2) |
| Psychiatric disorders | 26 (1.0) |
| Renal urinary tract disorders | 88 (3.4) |
| Respiratory disorders | 26 (1.0) |
| Rheumatologic disorders | 39 (1.5) |
| Skeletal disorders | 60 (2.3) |
| Tumor syndromes | 302 (11.8) |
| Total* | 2,560 (100) |

*Several participants have multiple diseases

**Supplementary Table 3** Overall frequency of SF genes according to the study groups

|  | Total | | Patient | | Family | | KoGES | |
| --- | --- | --- | --- | --- | --- | --- | --- | --- |
| **Cancer phenotypes** |  | |  | |  | |  | |
| Familial medullary thyroid cancer |  |  | |  | |  | |  |
| *RET* (1.0) | 3 | 1 | | 1 | | 1 | |  |
| Hereditary breast and/of ovarian cancer |  |  | |  | |  | |  |
| *BRCA1* (1.0) | 15 | 5 | | 6 | | 4 | |  |
| *BRCA2* (1.0) | 37 | 13 | | 17 | | 7 | |  |
| *PALB2* (3.0) | 6 | 3 | | 3 | |  | |  |
| Hereditary paraganglioma-phechromocytoma syndrome |  |  | |  | |  | |  |
| *SDHD* (1.0) | 1 |  | |  | | 1 | |  |
| *SDHC* (1.0) | 1 |  | | 1 | |  | |  |
| JPS |  |  | |  | |  | |  |
| *SMAD4* (2.0) | 1 | 1 | |  | |  | |  |
| Lynch syndrome (HNPCC) |  |  | |  | |  | |  |
| *MLH1* (1.0) | 7 | 1 | | 2 | | 4 | |  |
| *MSH2* (1.0) | 1 |  | | 1 | |  | |  |
| *MSH6* (1.0) | 6 |  | | 3 | | 3 | |  |
| *PMS2* (1.0) | 3 | 1 | | 1 | | 1 | |  |
| Neurofibromatosis type 2 |  |  | |  | |  | |  |
| *NF2* (1.0) | 1 | 1 | |  | |  | |  |
| PTEN hamartoma tumor syndrome |  |  | |  | |  | |  |
| *PTEN* (1.0) | 2 | 2 | |  | |  | |  |
| Tuberous selerosis complex |  |  | |  | |  | |  |
| *TSC1* (1.0) | 1 |  | | 1 | |  | |  |
| *TSC2* (1.0) | 6 | 2 | | 3 | | 1 | |  |
| **Cardiovascular phenotypes** |  |  | |  | |  | |  |
| Aortopathies |  |  | |  | |  | |  |
| *FBN1* (1.0) | 3 | 3 | |  | |  | |  |
| *TGFBR2* (1.0) | 1 |  | |  | | 1 | |  |
| *SMAD3* (1.0) | 1 |  | | 1 | |  | |  |
| *MYH11* (1.0) | 1 | 1 | |  | |  | |  |
| Arrhythmogenic right ventricular cardiomyopathy |  |  | |  | |  | |  |
| *PKP2* (1.0) | 3 | 1 | | 1 | | 1 | |  |
| *DSP* (1.0) | 2 | 1 | | 1 | |  | |  |
| *DSC2* (1.0) | 6 | 2 | | 2 | | 2 | |  |
| *DSG2* (1.0) | 7 |  | | 5 | | 2 | |  |
| Dilated cardiomyopathy |  |  | |  | |  | |  |
| *TNNT2* (1.0) | 10 | 5 | | 5 | |  | |  |
| *LMNA* (1.0) | 3 |  | | 2 | | 1 | |  |
| *FLNC* (3.0) | 2 |  | |  | | 2 | |  |
| *TTN* (3.0) | 49 | 20 | | 7 | | 22 | |  |
| Familial hypercholesterolemia |  |  | |  | |  | |  |
| *LDLR* (1.0) | 12 | 4 | | 3 | | 5 | |  |
| *APOB* (1.0) | 10 | 5 | | 4 | | 1 | |  |
| Hypertrophic cardiomyopathy |  |  | |  | |  | |  |
| *MYH7* (1.0) | 8 | 3 | | 5 | |  | |  |
| *MYBPC3* (1.0) | 10 | 2 | | 5 | | 3 | |  |
| *TNNI3* (1.0) | 9 | 3 | | 3 | | 3 | |  |
| *MYL3* (1.0) | 11 | 3 | | 4 | | 4 | |  |
| Long QT syndrome types 1 and 2 |  |  | |  | |  | |  |
| *KCNQ1* (1.0) | 4 | 1 | | 1 | | 2 | |  |
| *KCNH2* (1.0) | 5 | 1 | | 2 | | 2 | |  |
| Long QT syndrome 3, Brugada syndrome |  |  | |  | |  | |  |
| *SCN5A* (1.0) | 5 | 2 | | 2 | | 1 | |  |
| **Inborn errors of metabolism** |  |  | |  | |  | |  |
| Ornithine transcarbamylase deficiency |  |  | |  | |  | |  |
| *OTC* (2.0) | 2 | 1 | | 1 | |  | |  |
| **Miscellaneous phenotypes** |  |  | |  | |  | |  |
| Hereditary hemorrhagic telangiectasia |  |  | |  | |  | |  |
| *ACVRL1* (3.0) | 4 | 2 | | 2 | |  | |  |
| *ENG* (3.0) | 1 | 1 | |  | |  | |  |
| Malignant hyperthermia |  |  | |  | |  | |  |
| *RYR1* (1.0) | 36 | 13 | | 18 | | 5 | |  |
| *CACNA1S* (1.0) | 2 |  | |  | | 2 | |  |
| Total |  |  | |  | |  | |  |
| Genes | 41 | 30 | | 31 | | 25 | |  |
| Individual (%) | 280 (3.75) | 96 (4.39) | | 106 (3.80) | | 78 (3.12) | |  |

a

b

**Supplementary Fig. 1** Secondary finding rate according the ACMG SF phenotype (a) and gene (b) grouped by study

**Supplementary Fig. 2** Secondary findings in AR disorder related genes with heterozygous status

**Supplementary Table 4** Age, sex and lipid profile among the affected person with pathogenic variant of *LDLR* at baseline examination in KoGES cohort

| SF genes | Location/AA change | Variant class | Age | F_age | Sex | B_LIP | F_LIP | TCHL | TG | HDL-C | LDL-C | B_HTN | F_HTN |
| --- | --- | --- | --- | --- | --- | --- | --- | --- | --- | --- | --- | --- | --- |
| *LDLR* | Chr19:11102758-C-A (p.Cys95Ter) | Stop gained | 42 | 57 | M | Y | Y | 213 | 142 | 32 | 152 | Y | Y |
| *LDLR* | Chr19:11105567-G-A (p.Asp221Asn) | Missense | 48 | 65 | M | Y | Y | 265 | 177 | 36 | 193 | N | Y |
| *LDLR* | Chr19:11106666-G-A (p.Asp266Asn) | Missense | 62 | 78 | M | N | Y | 252 | 144 | 43 | 180 | N | Y |
| *LDLR* | Chr19:11113308-G-A (p.Arg406Gln) | Missense | 44 | 61 | M | N | Y | 225 | 79 | 52 | 157 | N | N |
| *LDLR* | Chr19:11116209-C-G (p.Leu568Val) | Missense | 48 | 64 | F | N | Y | 234 | 73 | 64 | 155 | N | Y |

B, baseline; F, final examination; LIP, hyperlipidemia; HTN, hypertension; TCHL, total cholesterol; TG, triglyceride; LDL-C, low-density lipoprotein cholesterol; HDL-C, High-density lipoprotein cholesterol; The LDL-C was calculated by LDL-C = TCHL - HDL - TG/5.

a

b

c

**Supplementary Fig. 3** Secondary finding rate using ClinVar database.
